# Design of a Negative Temperature Coefficient Temperature Measurement System Based on a Resistance Ratio Model

**DOI:** 10.3390/s24092780

**Published:** 2024-04-26

**Authors:** Ziang Liu, Peng Huo, Yuquan Yan, Chenyu Shi, Fanlin Kong, Shiyu Cao, Aimin Chang, Junhua Wang, Jincheng Yao

**Affiliations:** 1 State Key Laboratory of Functional Materials and Devices for Special Environmental Conditions, Xinjiang Key Laboratory of Electronic Information Materials and Devices, Xinjiang Technical Institute of Physics & Chemistry of CAS, 40-1 South Beijing Road, Urumqi 830011, China; liuziang21@mails.ucas.ac.cn (Z.L.); huopeng18@mails.ucas.ac.cn (P.H.); 107552103386@stu.xju.edu.cn (Y.Y.); shichenyu21@mails.ucas.ac.cn (C.S.); kfl1837388939@163.com (F.K.); caoshiyu22@mails.ucas.ac.cn (S.C.); changam@ms.xjb.ac.cn (A.C.); 2 Center of Materials Science and Optoelectronics Engineering, University of Chinese Academy of Sciences, Beijing 100049, China; 3 School of Physics Science and Technology, Xinjiang University, Urumqi 830046, China; 4 School of Physics and Materials Science, Changji University, Changji 831100, China

**Keywords:** temperature measurement system, NTC thermistor, resistance ratio

## Abstract

In this paper, a temperature measurement system with NTC (Negative Temperature Coefficient) thermistors was designed. An MCU (Micro Control Unit) primarily operates by converting the voltage value collected by an ADC (Analog-to-Digital Converter) into the resistance value. The temperature value is then calculated, and a DAC (Digital-to-Analog Converter) outputs a current of 4 to 20 mA that is linearly related to the temperature value. The nonlinear characteristics of NTC thermistors pose a challenging problem. The nonlinear characteristics of NTC thermistors were to a great extent solved by using a resistance ratio model. The high precision of the NTC thermistor is obtained by fitting it with the Hoge equation. The results of actual measurements suggest that each module works properly, and the temperature measurement accuracy of 0.067 °C in the range from −40 °C to 120 °C has been achieved. The uncertainty of the output current is analyzed and calculated with the uncertainty of 0.0014 mA. This type of system has broad potential applications in industry fields such as the petrochemical industry.

## 1. Introduction

Temperature, as an environmental factor, plays a critical role in various aspects of life, spanning from the industrial to agricultural domains [1]. Using a thermistor to sense the temperature, the system described here acts as a resistance bridge, collecting and processing the signal and transmitting it to the readout or control equipment. Due to the wide range of application scenarios and diverse needs, high demands are placed on precision temperature measurement equipment. As a result, achieving a high accuracy, wide temperature range, high stability, and low cost has become the focus of the development for modern equipment.

In previous research, the temperature measurement systems were designed based on temperature-induced material property changes, such as thermocouples, thermal resistors, and semiconductor sensors [2,3,4]. With advances in microprocessors, modern temperature measurement systems have been developed [5]. These devices convert continuous analog voltage and current signals into digitized data for further processing or storage. These advanced devices can utilize intricate calibration routines to enhance measurement precision, and they seamlessly integrate network connections for control, monitoring, and data transfer activities. Contemporary temperature measurement systems have additional features, such as process control and calibration maintenance. The calibration and maintenance features include automatic calibration, temperature compensation, remote monitoring, self-diagnostics, data logging, and so on. Shilei Du and Weiyan Li developed a module based on Freemodbus for smart temperature transmitters and validated its functionality [6]. Runze Mao et al. designed and manufactured a temperature measurement system that utilized multiple RTDs (Resistance Temperature Detectors) in parallel with capacitors. The RTDs in the system could be individually measured by impedance spectroscopy. The impedance information was then converted into a temperature signal, and the temperature measurement accuracy of this system was 0.7 K [7]. Kunming Zhao et al. designed a field-programmable gate array (FPGA) isolated transmitter using platinum resistors, which can be dynamically adjusted by an auto-correction algorithm to control current output [8]. The platinum resistors have remarkable chemical stability [9], high accuracy, and a wide operational temperature range [10]. However, the high costs and adverse reactions to high-vibration environments restrict the application of platinum resistors to complex industrial conditions [11]. Thermocouples are widely used for high-temperature measurements. The use of thermocouples is hindered by drawbacks such as low sensitivity and accuracy, susceptibility to noise, and susceptibility to corrosion [11,12]. Thermocouples are used with reference to junction compensation, which affects the use scenarios. Detailed characteristics of thermocouples, platinum resistors, and NTC thermistors are shown in Table 1 [13].

NTC thermistors are known for their high sensitivity, high robustness, fast response, and low cost. Hence, they are widely used in industry for temperature measurement, temperature monitoring, and inrush current suppression [14,15]. Romuald Masnicki and Dariusz Swisulski designed a multi-channel virtual device using NTC thermistors as temperature measuring elements. In this design, the NTC thermistor is calibrated using the Steinhart–Hart equation and the device has a measurement accuracy of 0.4 °C [16]. NTC thermistors are semiconductor ceramic elements made of transition metal oxides, whose resistance decreases with increasing temperature [17]. Transition metal oxides are usually metal oxides of Mn, Co, Fe, Zn, Ni, and other elements. In Equation (1), the relationship between the resistance and temperature of an NTC thermistor is depicted, known as the Hoge equation [18,19]:

(1) $$\frac{1}{T}=\sum_{i=0}^{n}{A_{i}(lnR)}^{n}\;\;\;(n\geq 2)$$

In this equation, *T* represents temperature, *R* represents resistance, and *A_i_* refers to equation parameters. With this equation, the NTC thermistor establishes a relationship between 1/*T* and *lnR* [18,20]. 

In order to leverage the high sensitivity and environmental stability of NTCs, and to perform temperature measurement across a wide temperature range, an NTC temperature measurement system based on the resistance ratio model was designed. The material constant *B* (K) plays a crucial role in characterizing the sensitivity of NTC thermal ceramic materials to temperature variations. Essentially, this parameter delineates the material’s responsiveness to temperature alterations and hence the degree of sensitivity exhibited towards the physical quantity being measured. As a rule of thumb, a higher material constant *B* implies greater sensitivity of the NTC thermal ceramic material to temperature fluctuations, thereby accentuating the material’s overall sensitivity. In practice, the material constant *B* is calculated by measuring the resistance at different temperatures, using Equation (2).

(2) $$B=\ln\left(\frac{R_{1}}{R_{2}}\right)/(\frac{1}{T_{1}}-\frac{1}{T_{2}})$$

where *R*_1_ and *R*_2_ are the resistances at temperatures *T*_1_ and *T*_2_, respectively. The resistance temperature coefficient α*_T_* refers to the change rate of the resistance value of the NTC thermistor for every 1 °C change in ambient temperature at zero load. α*_T_* is described by Equation (3), where *B* (K) is the material constant value and *T* (K) is the absolute temperature. Typically, NTC thermistors have a resistance temperature coefficient between −2%/°C and −6%/°C, which is higher than the 0.4%/°C of platinum resistors. Therefore, the temperature sensitivity of NTC thermistors is higher than that of platinum resistors.

(3) $$\alpha_{T}=-\frac{B}{T^{2}}$$

The challenges associated with NTC thermistors are vital to acknowledge alongside their evident advantages. One prominent issue lies in their nonlinear characteristics, posing a considerable hurdle for precise temperature measurements. Moreover, the heat generated during NTC thermistor operation impacts temperature readings, necessitating careful consideration in circuit design to prevent thermal issues. The power dissipation of NTC thermistors must also be carefully managed within the circuit to avert potential risks. Furthermore, the *B*-value of NTC thermistors plays a significant role in determining temperature measurement sensitivity. A higher *B*-value results in rapid resistance changes, thereby constraining the usable temperature range of NTC thermistors. Understanding these limitations is crucial for maximizing the effectiveness of NTC thermistors in temperature-sensing applications.

The inherent nonlinearity of NTC thermistors is a challenge [21], but the nonlinear characteristics of NTC thermistors can be mitigated through circuit design and programmatic interventions by using the resistance ratio model and precisely fitting the resistance–temperature equation of the NTC [22,23]. The purpose of this design is to give full play to the characteristics of high sensitivity and insensitivity to environmental vibration of the NTC thermistor and use it as a probe to build a temperature measurement system for industry. The system described in this paper achieved highly accurate temperature measurement over a wide range. Meanwhile, the circuit error of the system has been optimized, ensuring that the current output is maintained within the 4 to 20 mA range and that the loop current linearly correlates with the temperature. The design of the hardware temperature measurement circuit determines the accuracy of the resistance value acquisition. Error compensation, calibration equations, and digital filtering in the software affect the accuracy of temperature measurement. With the help of both, the designed system has improved in temperature measurement accuracy compared to previous designs. At the same time, the output current of the system has a low uncertainty. The combination of circuit design and software algorithms holds the promise of making the NTC thermistor a reliable temperature probe for industrial use.

## 2. Design and Experimental Section

### 2.1. Overall Design

**Figure 1:**
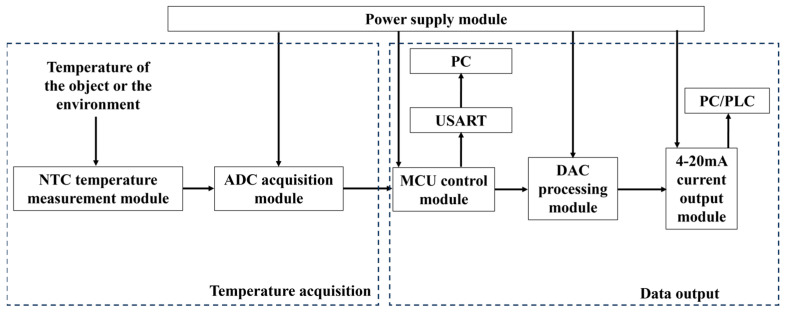
Block diagram of NTC thermistor temperature measurement system.

The basic framework of a temperature measurement system with the NTC thermistor is shown in Figure 1. Two critical components are involved: the temperature acquisition module and the transmission output module. The temperature acquisition module utilizes the NTC thermistor to collect environmental temperature data and transmit them to the data output module [24]. Here, the MCU calculates the resistance using Equation (1), and finally, the temperature is converted to a 4~20 mA current signal, which is recorded by the computer.

The NTC thermistor with a *B*_25/50_ value of 2100 K is selected to be used as the temperature probe in the temperature measurement system. The resistance value of the thermistor is 25 kΩ and 500 Ω at −40 °C and 120 °C approximately. The resistance temperature coefficient of the NTC thermistor is between −1.3%/°C and −3.8%/°C over this temperature range. The STM32F103 32-bit microcontroller is adopted as the central control chip, which is a standard 32-bit RISC processor underpinned by the ARMv7-M architecture with a Cortex-M3 core. The temperature values are calculated by embedded algorithms. The MCU is set to standby sleep mode to ensure that the device completes long-term continuous temperature measurements while using battery power. Operating in this mode effectively extends measurements and reduces the frequency of battery changes. In the loop current output module, the DAC chip AD421 is used as the core chip and the current value is controlled by the MCU by sending 16-bit binary data.

**Figure 2:**
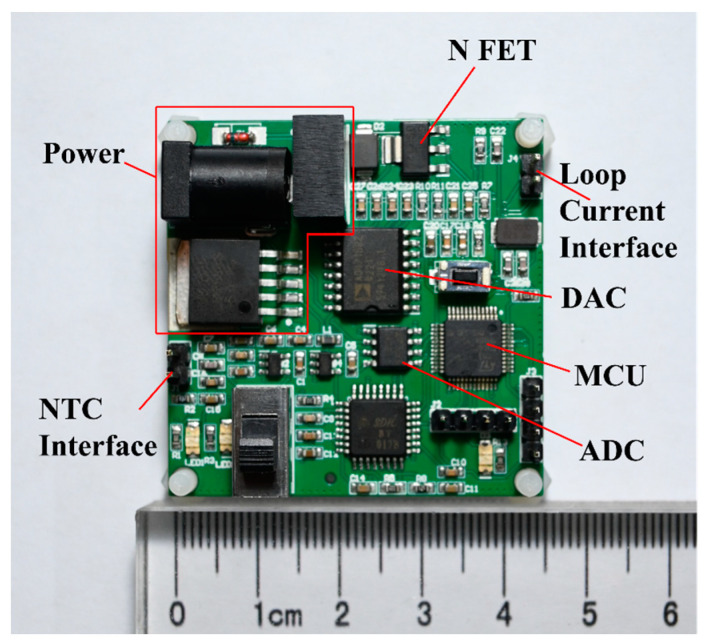
Manufactured PCBs for the temperature measurement system based on an NTC thermistor.

All components are integrated on a single printed circuit board (PCB), which minimizes the space required, improves system integration, and facilitates management and maintenance. The PCB of the NTC thermistor-based temperature measurement system is shown in Figure 2. The PCB is based on a two-layer structure and all components are mounted and soldered on the top layer. A DC power socket (6.4 mm OD and 2.0 mm ID) is located on the top of the board for external battery or voltage source power. In addition, two 2.54 mm 4-pin headers are available for programming, debugging, and data transfer to PCs. Two other 2.54 mm 2-pin headers are connected to the NTC thermistor and the output loop current. After soldering all components, the final dimension of the PCB is 45 mm × 45 mm × 10 mm.

### 2.2. Circuit Design

**Figure 3:**
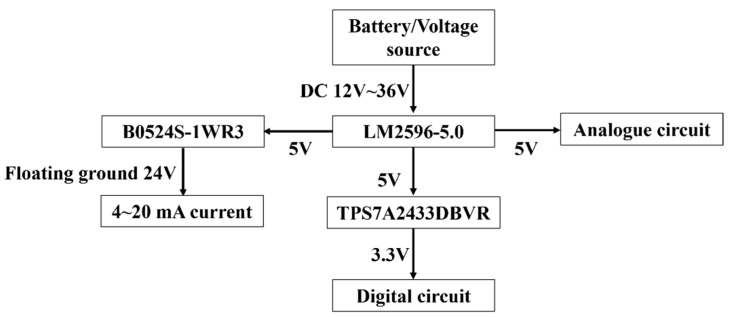
Schematic diagram of power supply for power module.

The power supply module design is a vital part of the temperature measurement system [25]. A stable, efficient, interference-proof, and reliable power supply design with low noise is a basic condition for the normal operation of the equipment. As shown in Figure 3, DC 12 V–36 V is adopted as the power source of the system. The power supply provides stable and clean DC power to ensure accurate and reliable operation. The LM2596 and TPS7A2433DBVR chips built into the system are used to generate 5 V and 3.3 V for the chips’ operation. Meanwhile, a fully isolated DC 24 V is applied for the loop current supply to guarantee the accuracy and stability of the output. The reference voltage of the temperature measurement circuit is filtered with multiple capacitors to reduce the impact of reference voltage noise on temperature measurement accuracy. At the same time, the digital power supply and analog power supply are isolated to avoid interference with the analog circuit.

**Figure 4:**
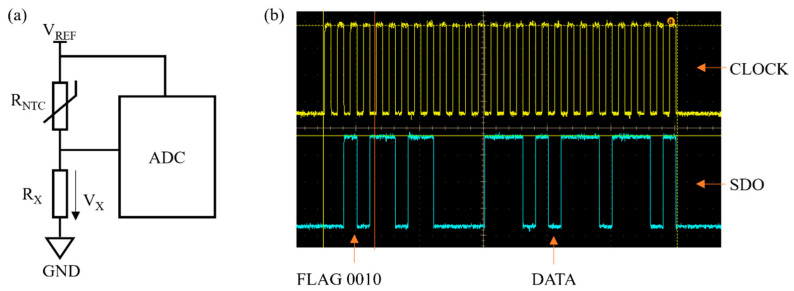
(**a**) Resistance ratio model; (**b**) oscilloscope acquisition signal diagram during ADC operation.

As shown in Figure 4a, the constant voltage source is used in the measurement circuit. A resistance ratio model is adopted, in which the thermistor, of resistance *R_NTC_*, is connected in series with a suitable voltage divider resistor (*R_X_* = 3300 Ω). In this way the acquisition voltage to the ADC does not exceed *V_REF_*, regardless of the value of *R_NTC_*. The thermal dissipation coefficient (δ) of the *NTC* thermistor used in the design has been tested to be approximately 2.51 mW/°C. The power of the thermistor is maximized when *R_NTC_* = *R_X_* = 3.3 kΩ, P_MAX_ = 0.1183 mW, and Δ*T* = P_MAX_/δ = 0.047 °C, which means the temperature rise at maximum power is 0.047 °C. In this design, the voltage of *V_REF_* is simultaneously delivered to the voltage divider and ADC reference [26], the inaccuracies attributed to reference voltage drift are eliminated, and the precision is enhanced. The voltage of the divider resistor is captured by the ADC, and the resistance of the *NTC* is calculated based on the voltage ratio model. The relationship between the resistance of the *NTC* (*R_NTC_*) and the voltage of the divider resistor (*V_X_*) is shown in Equation (4).

(4) $$V_{X}=V_{REF}*\frac{R_{X}}{R_{NTC}+R_{X}}$$

Therefore, the nonlinear relationship of resistance–temperature in *NTC* thermistors is mitigated. From the equation, *V_X_* is always less than *V_REF_*, i.e., the acquisition voltage is less than the reference voltage. The ADC oscilloscope acquisition diagram is presented in Figure 4b. In the data format described, the initial four bits (Bit31 to Bit28) serve as flag bits, with a specific configuration denoting different operational states. A flag bit sequence of ‘0010’ signifies the normal operation mode of the ADC. Following the flag bits, the subsequent 24 bits (Bit27 to Bit4) represent the acquired acquisition data, constituting the core information for processing and analysis within the system. It is essential to recognize that the final four bits (Bit3 to Bit0) hold no practical value and are random numbers without functional relevance. These bits are omitted from the representation to streamline the data visualization process, focusing attention on the critical flag and acquisition data segments. It can be observed that the data of the first 4 bits comply with the flag bits, and then the subsequent 24 bits are acquired and calculated as the *V_X_*. However, the wire resistance on the *NTC* thermistor still exists due to the resistance ratio model. The *NTC* thermistor adopts the two-wire connection method. The total length of the wires connecting the *NTC* thermistor is 2 m. The wire used for the test has a resistance of 153 Ω for 1 km. This results in a resistance measurement error of 0.306 Ω. In the measurement, the *NTC* thermal probe is placed in the environment to be measured while the wire is at room temperature. Therefore, when calculating the error of the wire, resistance is considered to be a constant value. Based on the fitting of the Hoge-2 equation for the *NTC* thermistor, the wire resistance is calculated to cause a temperature measurement error of 0.038 °C when making measurements at 120 °C. Wire resistance is introduced as a variable in the system to solve the issue. To mitigate the effects of wire errors on the temperature measurement, the resistance is entered by the operator based on the length of the wire.

**Figure 5:**
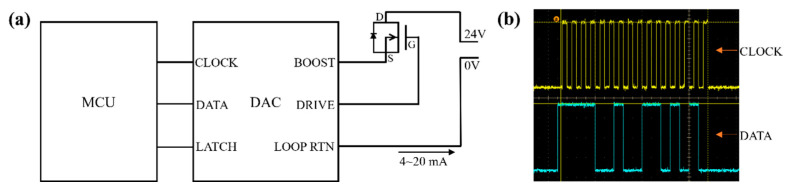
(**a**) DAC circuit schematic; (**b**) oscilloscope acquisition signal diagram during DAC operation.

Figure 5a is a schematic diagram of the loop current output. A N-type field effect transistor (FET) is connected between the AD421 controller chip and the loop current output unit. The FET is a voltage-controlled device whose conduction and current are controlled by the voltage at the DRIVE pin. As shown in Figure 5b, the current is dictated by a 16-bit binary number, which denotes the temperature transmitted from the MCU to the AD421 chip in the program. 

Loop-powered transmitters as well as the peripheral components have stringent power consumption requirements, and low power consumption is an important parameter. The MCU processor is kept in a low-power state by reducing unnecessary outputs and loads. The peripheral circuitry operates at less than 3.25 mA [27]. With this precaution, the accuracy of the loop current output is not affected by peripheral circuitry. When the loop current value is small, e.g., 4 mA, the design creates a stable current and improves current accuracy [28]. If the NTC thermistor is replaced with another model, the range of the resistance value changes. For the resistor ratio model, the divider resistor and the reference voltage are re-selected. The goal is to keep the ADC’s acquisition voltage in a reasonable range interval. The DAC and other chips can be left unchanged, and only the calibration equations and current correspondences need to be rewritten in the software.

### 2.3. Experimental Program

**Figure 6:**
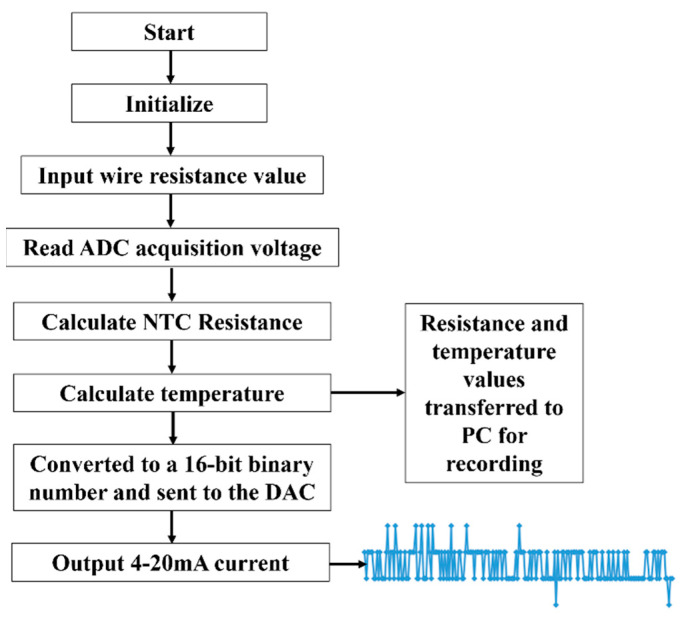
The flow chart of system processing.

A flowchart of the system for processing and transmitting temperature measurement data is shown in Figure 6. The waveforms of the data pins and clock pins of the ADC and DAC are measured by the oscilloscope. Compare the consistency of the acquired waveforms with the data collected by the PC to determine proper data transfer and proper module operation. The calculated resistance and temperature values are used as intermediate process variables, which are transmitted by the MCU through the USART serial port to the host computer for monitoring and saving. The current values are captured by the ammeter and transferred to the measurement system for recording.

The precision of temperature measurement depends on the accuracy of the resistance measurement. Firstly, stability and reliability are crucial to the measurement system. Secondly, the accuracy of the system is verified by continuously sampling the DC resistor box, and the error is corrected by the error function.

The precise calibration of the R-T relationship of NTC thermistors is the basis for the temperature measurement function. The standard platinum resistance thermometer is used to monitor the temperature in a constant-temperature environment, and the monitored temperature is used as the actual temperature. The constant-temperature oil tank used is produced by Fluke. Its environmental stability is less than ±0.0015 °C and uniformity is less than ±0.003 °C. The standard platinum resistance thermometer is used to monitor the temperature of the constant-temperature oil bath, and the experimental measurement is carried out half an hour after it reaches the set temperature to ensure the effectiveness and repeatability of the experimental results. The simultaneous acquisition of the NTC resistor and platinum resistor temperatures is established to create a Hoge equation. This equation is used by the software program to calculate the temperature measured by the temperature measurement system.

A DC resistor box with an accuracy of 0.01% is plugged into the system and the calculated temperature value is received by the PC. The current waveform is observed to verify that the output is stable. Finally, the NTC thermistor is accessed as a temperature probe and a platinum resistor is used to monitor the constant temperature environment. The output current is measured and recorded, and the corresponding temperature is calculated. The temperature measurement error is calculated by comparing this temperature with the platinum resistance monitoring temperature.

## 3. Results

**Figure 7:**
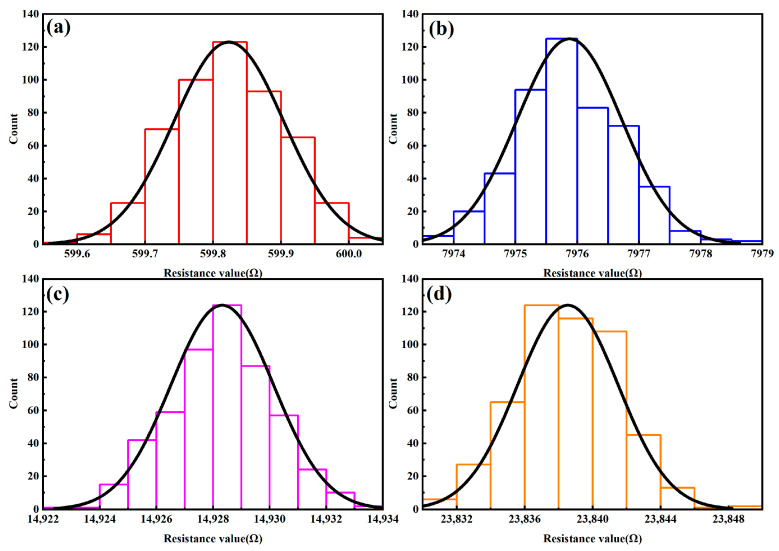
Distribution of sampling value: (**a**) 0.6 kΩ, (**b**) 8 kΩ, (**c**) 15 kΩ, and (**d**) 24 kΩ.

A standard resistance box was connected to the measurement system. Tests with the resistor box were performed in a stable environment at 25 °C ± 1 °C. This prevents the effect of temperature changes on the resistance value of the resistance box. The accuracy class of the resistor box used in this experiment is 0.01 and the maximum error of the resistor box is 0.01% of the selected resistance value. Over 5000 data collections were performed for each data point. A median average filtering algorithm was used for digital filtering. Every ten data points are grouped together, the maximum and minimum values are discarded, and the arithmetic mean of the remaining eight data points is used as the final measurement data. For the resistances of 0.6 kΩ, 8 kΩ, 15 kΩ, and 24 kΩ, the collection results are shown in Figure 7, and the sampled values are normally distributed, indicating that the collected resistance value data are reliable.

**Figure 8:**
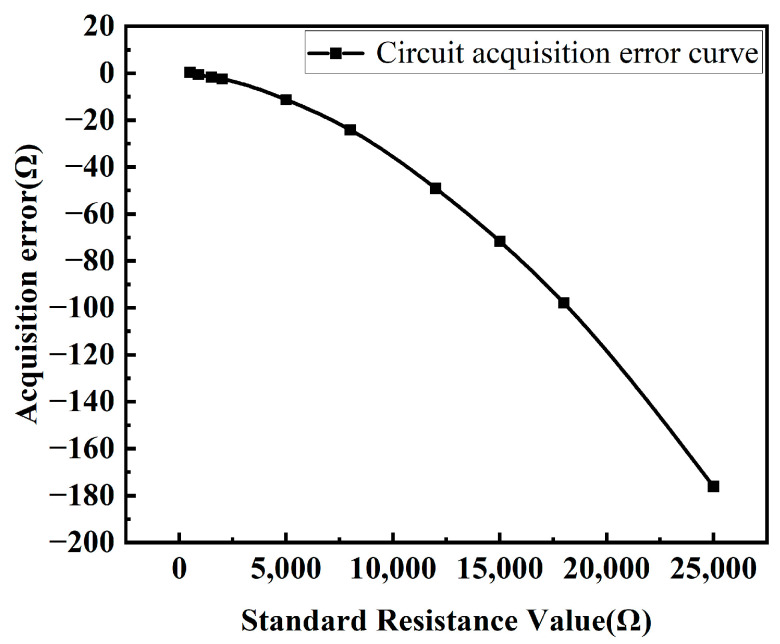
Standard resistance vs. acquisition error curve.

The measurement system was used to collect data from ten different standard resistance values to verify the stability of the collected values and to determine the collection errors. The error values are shown in Figure 8. As can be seen from the figure, there is a clear quadratic relationship between the acquisition error and the measured standard resistance.

A linear function relationship $R_{2}=P_{1}\times {R_{1}}^{2}+P_{2}\times R_{1}+P_{3}$ is established between the system’s acquisition of resistance values and the standard resistance values. The coefficients are *P*_1_ = 2.256446 × 10^−7^, *P*_2_ = 1.001535, and *P*_3_ = −1.135937. A total of 5000 acquisitions are made for each standard fixed-value resistor, where the fixed-value resistor is *R*. The system-measured resistance value is *R*_1_. The average value of the system-acquired resistance after the introduction of the error is *R*_2_, and the error value is Δ*R*_2_. The temperature error calculated from Δ*R*_2_ is Δ*T*. The standard deviation of *R*_2_ as well as the coefficient of variation are shown in Table 2. From the standard deviation in the table, it can be seen that the measurement of the resistance value is stable, and the coefficient of variation indicates that the difference between the collected resistance value and the standard resistance is small. With the introduction of the error equation, the temperature measurement error due to the sampling error value is very small and does not affect the subsequent temperature measurement.

The Hoge equation was employed to perform polynomial fitting on 1/*T* and *lnR* to achieve higher precision. Considering the calibration equations’ fitting accuracy and the system’s computational complexity, *n* = 3, i.e., the Hoge-2 equation, was chosen for fitting in the calibration equations [29,30,31]. Its fundamental form is described by Equation (5):

(5) $$\frac{1}{T}=A_{0}+A_{1}\times lnR+A_{2}\times ({lnR)}^{2}+A_{3}\times ({lnR)}^{3}$$

**Figure 9:**
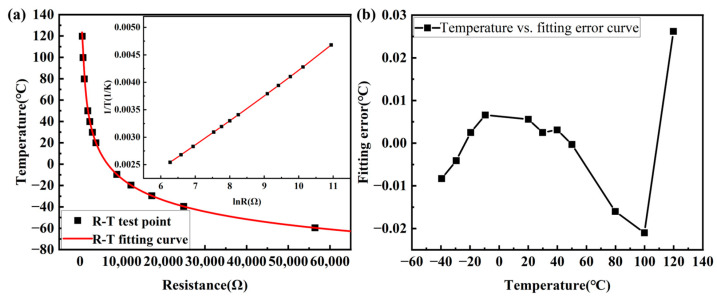
(**a**) *R* vs. *T* curve and *lnR* vs. 1/*T* curve; (**b**) *T* vs. fitting error curve.

In this equation, *T* (K) represents temperature, *R* (Ω) represents resistance, and *A_i_* refers to equation parameters. The parameters are *A*_0_ = −2.454812 × 10^−4^, *A*_1_ = 4.874768 × 10^−4^, *A*_2_ = −1.132064 × 10^−5^, and *A*_3_ = 7.250193 × 10^−7^. SSE (the sum of squares due to error) is 1.192 × 10^−13^, *R*-square (coefficient of determination) is 1, adjusted *R*-square (degree-of-freedom adjusted coefficient of determination) is 1, and RMSE (Root Mean Square Error) is 1.221 × 10^−7^. The test points and the fitted curves are shown in Figure 9a. It shows that the resistance value of the NTC thermistor decreases sharply with increasing temperature, and the fitted curves are well fitted to the test points. The fitting error is shown in Figure 9b, and the maximum fitting error occurs at a temperature value of 120 °C, with the error value of 0.0262 °C. The fitting error of the Hoge-2 equation is very small, and the effect of nonlinearity in the *R*-*T* curve of the NTC thermistor is reduced by this fitting.

**Figure 10:**
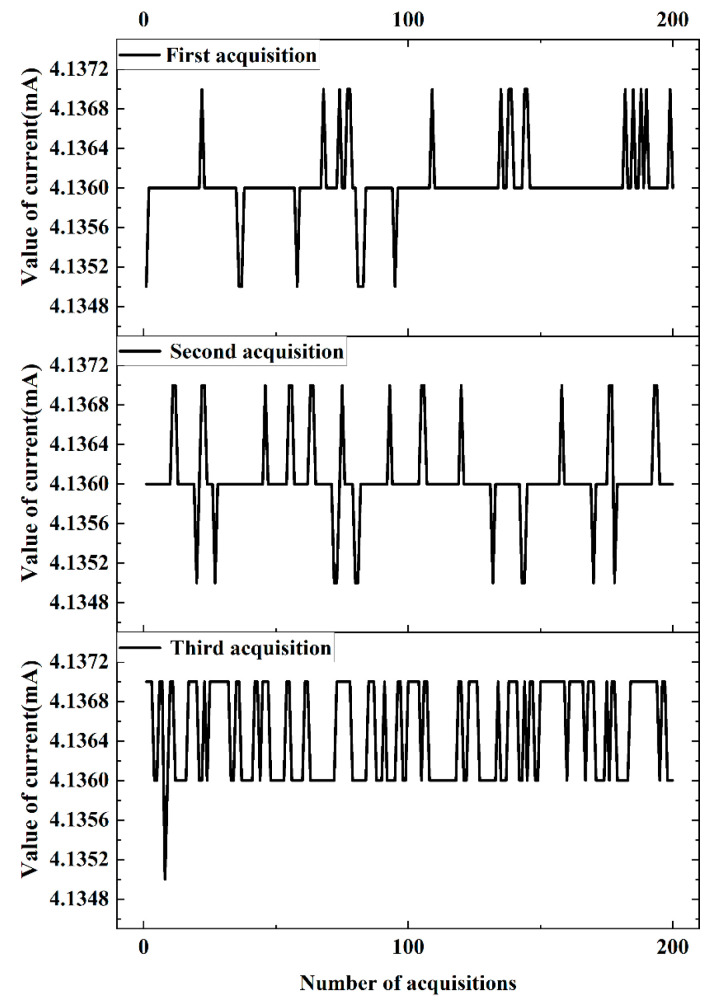
Temperature measurement system current output test.

In order to verify the accuracy and stability of the current output, the output value of the circuit loop was measured with the standard resistor box instead of the NTC thermistor. Repeat experiments were conducted using the 24,000 Ω resistor connected to the temperature measurement system, and the current value of the data output was collected by an ammeter. The ammeter used for testing was an Agilent 34411A from USA. It is a 6.5-bit resolution digit multimeter. When it is connected to a computer via LAN or USB, current data are read and recorded by the computer. As can be seen in Figure 10, the current fluctuation is small, and the temperature measurement system output is stable. The error between the collected current value converted to the temperature value and the temperature value collected from the PC is less than 0.02 °C, which indicates the high accuracy of the current output.

**Figure 11:**
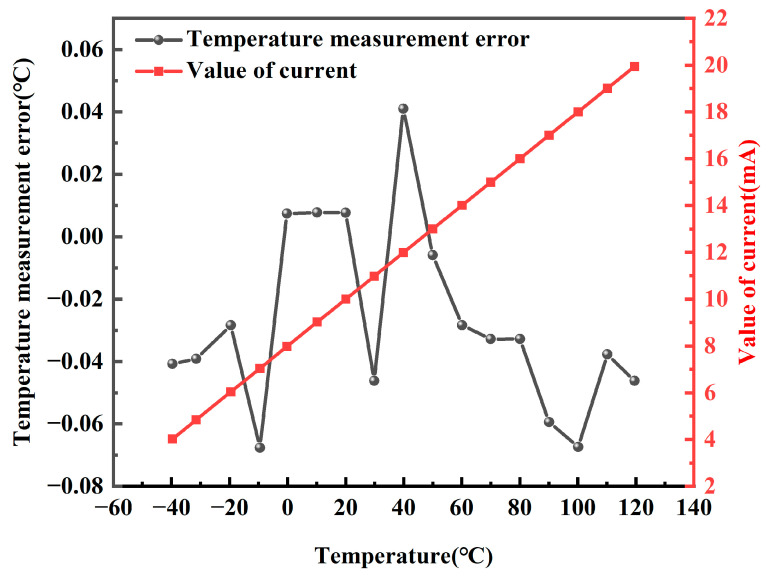
The trend of current output and temperature measurement error at different temperatures.

NTC thermistors were integrated into the system as temperature probes. The thermostatic oil baths Fluke 7080, Fluke 7012, and Fluke 6020 from USA were used to provide thermostatic environments with an accuracy of 0.003 °C over the temperature range of −40 °C to 120 °C. The standard platinum thermistor PT25 was used in conjunction with the Fluke 1595A for monitoring thermostatic environments. System measurements were carried out in the range from −40 °C to 120 °C at 10 °C intervals. The current output values of the system were collected and converted to temperature values to calculate the measurement error of the system. The temperature measurement error and output current of the system are shown in Figure 11. The maximum measurement error is −0.067 °C at 100 °C. The measured current is linear with respect to temperature, and the current stability is excellent.

**Figure 12:**
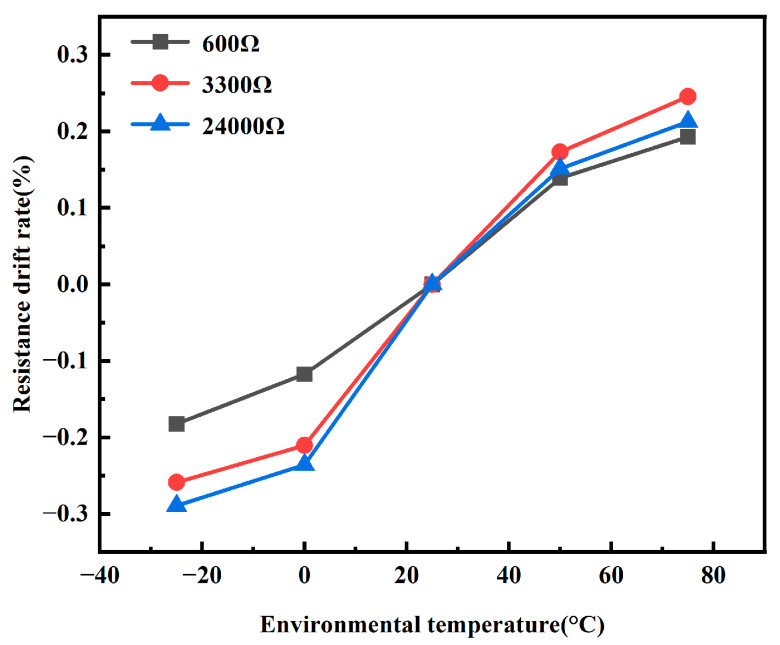
Resistance measurement of drift rate in relation to temperature.

Typically, the temperature probe is located in the environment being measured and the temperature measurement system is located in a room-temperature environment. The systems are also used in extreme environments, so environmental testing was performed on the PCB. The PCB was placed in different ambient temperatures to perform the resistance acquisition of the resistance box. The PCB does not function properly in environments below −40 °C and above 100 °C. The collected resistance at a room temperature of 25 °C was used as the standard value and the resistance drift rate was calculated using the collected values at other temperatures. The result of the resistance drift rate calculation is shown in Figure 12. The maximum resistance drift rate is 0.30%. The temperature measurement error is calculated from the resistance drift rate. The maximum error occurs when the 600 Ω resistance box is tested in a 75 °C environment. The error value was 0.122 °C (0.015%/10 °C).

The mathematical model for uncertainty analysis is shown in Equation (6).

(6) $$\Delta I=I_{d}-[\frac{I_{m}}{t_{m}}\left(t_{s}-t_{0}\right)+I_{0}]$$

where Δ*I* is the measurement error of the temperature measurement system at temperature t and *I_d_* is the output current value. *I_m_* is the output range of the temperature measurement system. *t_s_* is the input temperature value, and *t_m_* is the input range of the system. *t*_0_ is the lower temperature value of the input and *I*_0_ is the theoretical lower limit of the output current. The temperature measurement system has a measuring range of −40 °C to 120 °C and a current output range of 4 to 20 mA. The formulae for the sensitivity coefficients *C*_1_ and *C*_2_ are shown in Equations (7) and (8).

(7) $$C_{1}=\frac{\partial \Delta I}{\partial I_{d}}=1$$

(8) $$C_{2}=\frac{\partial \Delta I}{\partial t_{s}}=-\frac{I_{m}}{t_{m}}=-\frac{16}{160}=-0.1\;(mA/{}^{\circ}C)\;$$

The source of uncertainty of the input quantity *I_d_* has two components, the repeatability of the output current of the measured system and the measurement error of the electrical measuring instrument. The temperature measurement system repeatability test data are shown in Table 3.

The uncertainty due to the repeatability of the output current is *u*(*I_d_*_1_). Data were adjudicated by using the extreme variance method. The calculation of *u*(*I_d_*_1_) is shown in Equations (9)–(12).

(9) $$0\;{}^{\circ}C:\;s\left(I_{d1}\right)=\frac{7.976-7.974}{1.69}=0.0012\;(mA)$$

(10) $$u\left(I_{d1}\right)=\frac{0.0012}{\sqrt{6}}=0.0005\;(mA)$$

(11) $$50\;{}^{\circ}C:\;s\left(I_{d1}\right)=\frac{12.999-12.997}{1.69}=0.0012\;(mA)$$

(12) $$u\left(I_{d1}\right)=\frac{0.0012}{\sqrt{6}}=0.0005\;(mA)$$

From the calculations, it can be seen that the standard uncertainty due to the repeatability error of the two temperature points is equal. Therefore, the value of *u*(*I_d_*_1_) is 0.0005 mA. The uncertainty introduced by the measurement error of the ammeter is *u*(*I_d_*_2_). The multimeter used for this test was an Agilent 34411A, which has a current resolution of 1 μA. Its current indication error does not exceed ±0.5 μA. *u*(*I_d_*_2_) is calculated as Equation (13) according to the uniform distribution estimation.

(13) $$u\left(I_{d2}\right)=\frac{0.0005}{\sqrt{3}}=0.0003\;(mA)$$

*u*(*I_d_*_1_) and *u*(*I_d_*_2_) are independent of each other, so *u*(*I_d_*) is calculated by Equation (14).

(14) $$u\left(I_{d}\right)=\sqrt{{u\left(I_{d1}\right)}^{2}+{u\left(I_{d2}\right)}^{2}}=0.0006\;(mA)$$

The uncertainty introduced by the input quantity *t_s_* is *u*(*t_s_*), which mainly originates from the temperature measurement error of the first-class platinum resistor and the influence of fluctuations in the thermostat bath. The uncertainty error *u*(*t_s_*_11_) for the repeatability of platinum resistance measurements is calculated in Equations (15)–(18).

(15) $$0\;{}^{\circ}C:s\left(t_{s11}\right)=\frac{-0.263-(-0.267)}{1.69}=0.0024\;({}^{\circ}C)$$

(16) $$u\left(t_{s11}\right)=\frac{0.0024}{\sqrt{6}}=0.0010\;({}^{\circ}C)$$

(17) $$50\;{}^{\circ}C:s\left(t_{s11}\right)=\frac{49.981-49.978}{1.69}=0.0018\;({}^{\circ}C)$$

(18) $$u\left(t_{s11}\right)=\frac{0.0018}{\sqrt{6}}=0.0007\;({}^{\circ}C)$$

As can be seen from the calculations, the uncertainty calculated for the 0 °C repeatability error is large. The value of *u*(*t_s_*_11_) is 0.0010 mA. In temperature monitoring, first-class platinum resistors are used, whose self-heating effect introduces uncertainty. A search of the certificates shows that the self-heating effect of platinum resistors is 1.9 mK. Since this effect is widespread, the self-heating effect can be regarded as a two-point distribution. The uncertainty error *u*(*t_s_*_12_) due to the self-heating effect of platinum resistance is shown in Equation (19).

(19) $$u\left(t_{s12}\right)=\frac{0.0019}{1}=0.0019\;({}^{\circ}C)$$

The long-term stability of the platinum resistor is less than 2 mK. Considered to be uniformly distributed, the standard uncertainty *u*(*t_s_*_13_) introduced is calculated as Equation (20).

(20) $$u\left(t_{s13}\right)=\frac{0.002}{\sqrt{3}}=0.001\;({}^{\circ}C)$$

The uncertainty introduced by the accuracy of the standard platinum resistor is *u*(*t_s_*_14_). The standard platinum resistor has an accuracy of less than 2.4 mK, and *u*(*t_s_*_14_) is calculated as a uniform distribution in Equation (21).

(21) $$u\left(t_{s14}\right)=\frac{0.0024}{\sqrt{3}}=0.0014\;({}^{\circ}C)$$

*u*(*t_s_*_11_), *u*(*t_s_*_12_), *u*(*t_s_*_13_), and *u*(*t_s_*_14_) are independent of each other. The uncertainty *u*(*t_s_*_1_) introduced by the platinum resistance is calculated as Equation (22).

(22) $$u\left(t_{s1}\right)=\sqrt{{u\left(t_{s11}\right)}^{2}+{u\left(t_{s12}\right)}^{2}+{u\left(t_{s13}\right)}^{2}+{u\left(t_{s14}\right)}^{2}}=0.0028\;({}^{\circ}C)$$

The uncertainty of the temperature inhomogeneity of the thermostatic oil bath is *u*(*t_s_*_2_). The maximum temperature difference of the thermostatic oil bath is 0.003 °C. Considering it as a uniform distribution, *u*(*t_s_*_2_) is calculated as Equation (23).

(23) $$u\left(t_{s2}\right)=\frac{0.003}{\sqrt{3}}=0.0017\;({}^{\circ}C)$$

The uncertainty of the temperature fluctuation of the thermostatic oil bath is *u*(*t_s_*_3_). The stability of the thermostatic oil bath is 0.0015 °C. Considering it to be uniformly distributed, *u*(*t_s_*_3_) is calculated in Equation (24).

(24) $$u\left(t_{s3}\right)=\frac{0.0015}{\sqrt{3}}=0.0009\;({}^{\circ}C)$$

*u*(*t_s_*_1_), *u*(*t_s_*_2_), and *u*(*t_s_*_3_) are independent of each other. The uncertainty *u*(*t_s_*) introduced by the input t_s_ is calculated as Equation (25).

(25) $$u\left(t_{s}\right)=\sqrt{{u\left(t_{s1}\right)}^{2}+{u\left(t_{s2}\right)}^{2}+{u\left(t_{s3}\right)}^{2}}=0.0034\;({}^{\circ}C)$$

The standard uncertainty components analyzed above are uncorrelated, so the relative standard uncertainty *u_c_*(Δ*I*) is calculated as Equation (26).

(26) $$u_{c}\left(\Delta I\right)=\sqrt{{{|C}_{1}|}^{2}{u\left(I_{d}\right)}^{2}+{{|C}_{2}|}^{2}{u\left(t_{s}\right)}^{2}}=0.0007\;(\mathrm{mA})$$

When calculating the extended uncertainty, the temperature measurement system is checked to *k* = 2. The extended uncertainty *U_k_*_=2_ is shown in Equation (27).

(27) $$U_{k=2}=ku_{c}\left(\Delta I\right)=0.0014\;(mA)$$

The test results show that the accuracy of temperature measurement is 0.067 °C and the uncertainty is 0.014 °C. The temperature measurement reliability and accuracy of the constructed system are demonstrated, thus further proving the validity of the design methodology. The designed temperature measurement system accurately measures air and oil in the laboratory. Similarly, this temperature measurement system can be applied to the precise temperature measurement in buildings, petroleum, chemical, and other industrial temperature measurement fields.

The iTEMP TMT127 DIN rail temperature transmitter from E+H, for example, has a temperature measurement accuracy of 0.2 °C from −50 °C to 200 °C and costs USD 200. For the designed temperature measurement system, the temperature measurement accuracy of 0.04%FS was achieved, which is better than the 0.08%FS of the temperature transmitter described above. In terms of cost, this design is about USD 55, which is a reduction in cost compared to its counterpart. For maintenance, the design is highly integrated and has no redundancy. NTC thermistor probes are highly stable with low ageing drift. Only regular calibration and maintenance of the PCB is required.

The temperature measuring system is designed without pressure- and vibration-related environmental tests. In subsequent iterations, the corresponding experiments should be supplemented to eliminate the influence of pressure and vibration environment on measurement accuracy. In subsequent developments, the temperature measurement system can be combined with additional fieldbus protocols to extend its functionality for communication and calibration maintenance. This will essentially mean that these devices will not only record temperature but also analyze, anticipate, and communicate these data to other devices or centralized systems in real-time.

## 4. Conclusions

In summary, the design of a temperature measurement system based on NTC thermistors has been accomplished. The temperature measurement accuracy is less than 0.067 °C in the temperature range of −40 °C to 120 °C, which has been improved by the circuit’s linear error correction. The uncertainty of the current output is 0.0014 mA. The temperature measurement system demonstrates high accuracy and stability. The designed resistance ratio model significantly reduces the effects of power supply drift and mitigates the impact of the nonlinearity of NTC thermistors on voltage acquisition. The wire resistance can be adjusted manually by the field operator. This method effectively mitigates the effect of wire resistance on temperature measurement. Simultaneously, the results of temperature tests confirm the practical effectiveness of the system. By harnessing the emerging technologies, temperature measurement systems are able to perform better in areas such as NTC calibration, device communication, and equipment maintenance. It will greatly facilitate its application in industry and improve the accuracy of temperature measurements.

## Figures and Tables

**Table 1:** Characteristics of temperature sensors.

Characteristic	Thermocouples	Platinum Resistors	NTC Thermistors
Materials Used	Different metal combinations	Platinum (wire or film)	Semiconductor materials
Change Parameters	Voltage	Resistance	Resistance
Temperature Range	−200 °C to 2300 °C (depending on type)	−200 °C to 850 °C	−200 °C to 1000 °C
Sensitivity	1–80 mV/°C	0.385%/°C (for Pt100)	−2%/°C~−6%/°C
Response Time	Fast 0.1–10 s	Slow 1–50 s	Fast 0.1–10 s
Stability	Moderate	Excellent	Moderate
Cost	Low	High	Low to Moderate
Advantages	Wide temperature range, rugged, low cost	High accuracy, linear response, stable	High sensitivity, small size, low cost
Disadvantages	Nonlinear response, drift over time, cold end compensation, low sensitivity	Slow response time, higher cost, sensitive to shock	Nonlinear response, limited temperature range

**Table 2:** Error and standard deviation after introduction of error function.

*R* (Ω)	*R*_1_ (Ω)	*R*_2_ (Ω)	Δ*R*_2_ (Ω)	Standard Deviation of Measured Values	Variation Coefficient (%)	Δ*T* (°C)
500	500.347	500.036	0.037	0.0314	0.0063	−0.0050
900	899.388	899.916	−0.084	0.0945	0.0105	0.0052
1500	1498.34	1500.01	0.011	0.1228	0.0082	−0.0003
2000	1997.5	2000.34	0.335	0.2212	0.0111	−0.0075
5000	4988.7	5000.84	0.842	0.3870	0.0077	−0.0061
8000	7975.87	8001.34	1.336	0.8518	0.0107	−0.0055
12,000	11,950.9	12,000.3	0.331	1.2447	0.0104	−0.0008
15,000	14,928.3	15,000.4	0.391	1.8363	0.0122	−0.0007
18,000	17,902.1	18,000.8	0.781	2.0814	0.0116	−0.0012
25,000	24,823.9	25,000	−0.048	1.1747	0.0047	0.0001

**Table 3:** Temperature measurement system repeatability test data.

Checked Temperature (°C)			Output Value (mA)	Platinum Resistance Temperature Value (°C)
0	1	Upstroke	7.974	−0.265
		Downstroke	7.975	−0.264
	2	Upstroke	7.974	−0.265
		Downstroke	7.976	−0.263
	3	Upstroke	7.975	−0.265
		Downstroke	7.976	−0.267
	Average value		7.975	7.975
50	1	Upstroke	12.997	49.979
		Downstroke	12.998	49.978
	2	Upstroke	12.997	49.980
		Downstroke	12.998	49.978
	3	Upstroke	12.997	49.979
		Downstroke	12.999	49.981
	Average value		12.997	12.997

## Data Availability

Data are contained within the article.
